# OpenMTA, a paradigm shift in exchanging biological material

**DOI:** 10.1093/synbio/ysy021

**Published:** 2018-12-05

**Authors:** Konstantinos Vavitsas

**Affiliations:** 1 Australian Institute of Bioengineering and Nanotechnology, The University of Queensland, Brisbane, QLD 4072, Australia; 2 CSIRO Synthetic Biology Future Science Platform, GPO Box 2583, Brisbane, QLD 4001, Australia

Collaborative research requires the regular exchange of ideas, knowledge and often samples and materials. Nowadays, all types and volumes of information flow unconstrained by geographical location, but the exchange of biological materials follows different norms.

One of the limiting factors in obtaining samples from a research institution is signing a Material Transfer Agreement (MTA). The process is time-consuming and nonuniform, as each organization has their own preferred terms and conditions. Some typical terms specify the permitted use of the material, may forbid the distribution of derivatives, and generally prohibit commercial applications. There are a handful of commonly accepted MTAs available, such as the Uniform Biological Material Transfer Agreement (UBMTA) (ott.nih.gov/resources). Even though UBMTA provides standard terms, it still prohibits commercial uses and redistribution of derivatives. This particularly hinders the commercialization of ideas, even in cases where researchers and students want to pursue inventions they themselves developed.

The recently published OpenMTA (openmta.org) addresses the limitations of existing MTAs. It intends to facilitate the exchange of material by providing flexibility to researchers and institutions on how their material can be shared, while still addressing security and legal concerns that traditional MTAs cover (1). The development of the OpenMTA was a collaborative effort between the BioBricks Foundation and the OpenPlant Synthetic Biology Research Centre. The goal of the initiative was to create a legal framework that can be used in an international context, allowing the recipient to use the material for any lawful purpose and permitting redistribution. Each OpenMTA includes a master agreement that cannot be edited and an implementing letter that can be customized to allow users to agree on terms such as attribution, whether further transfers must be reported back to the originator, and payment of processing fees suitable for a specific transaction. Research organizations can review and become signatories of the Master Agreement in order to give their employees this new option. Importantly, institutions that adopt the Master Agreement do not have to use it for all transfers and can customize based on the specific material being transferred.

The OpenMTA covers a crucial need for synthetic biology, a discipline which relies on recombining biological building blocks available from various community resources such as the iGEM registry or Addgene. The synthetic biology community generally adheres to principles of openness (opendefinition.org), but it lacked the tool needed to apply this philosophy to the exchange of biological material.

Interestingly, the exchange of biological material between gene synthesis companies and users of synthetic DNA does not involve the execution of an MTA because, in this case, the provider—DNA Synthesis Company—does not claim ownership of the biological material they produce. While well-resourced institutes and companies can overcome the need for MTAs by DNA synthesis, such a strategy is cost prohibitive for many start-ups and young group leaders. As the cost of synthetic DNA decreases, it may reach the point where it becomes cheaper to synthesize DNA molecules than it is to store them. In that case, the free exchange in the synthetic biology community may become less dependent on the MTA regulating the transfer of DNA molecule and more dependent on the wide adoption of open copyright licenses to protect DNA or protein sequences (creativecommons.org). Until then, OpenMTA could be an important legal tool enabling synthetic biologists to address real-world problems and have a positive contribution to society.
